# The impact of a history of child abuse on cognitive performance: a cross-sectional study in older patients with a depressive, anxiety, or somatic symptom disorder

**DOI:** 10.1186/s12877-022-03068-6

**Published:** 2022-04-28

**Authors:** F. M. Tjoelker, H. W. Jeuring, I. Aprahamian, P. Naarding, R. M. Marijnissen, G. J. Hendriks, D. Rhebergen, A. Lugtenburg, M. W. Lammers, R. H. S. van den Brink, R. C. Oude Voshaar

**Affiliations:** 1 University of Groningen, University Medical Center Groningen, Rob Giel Research Center (RGOc), PO Box 30.001, 9700 HB Groningen, The Netherlands; 2 Group of Investigation on Multimorbidity and Mental Health in Aging (GIMMA), Geriatrics Division, Internal Medicine Department, Jundiaí Medical School, Jundiaí, Brazil; 3 GGNet Mental Health, Division of Old Age Psychiatry, Warnsveld & Apeldoorn, The Netherlands; 4 Behavioural Science Institute, Radboud University & Pro Persona Institute for Integrated Mental Health Care, Nijmegen, The Netherlands; 5 Mental Health Center GGZ Centraal, Ermelo, The Netherlands & Psychiatry, Amsterdam UMC, VU University Medical Center, Amsterdam Public Health Research Institute, Amsterdam, Netherlands; 6 Mental Health Center GGZ Drenthe, Assen, The Netherlands; 7 Mediant Mental Health Center, Enschede, The Netherlands

**Keywords:** Cognition, Cognitive performance, Adverse childhood experiences, Child abuse, Mood disorders, Anxiety disorders, Somatic symptom disorders, Aged, Mental health

## Abstract

**Background:**

Child abuse is a major global burden with an enduring negative impact on mental and physical health. A history of child abuse is consistently associated with worse cognitive performance among adults; data in older age groups are inconclusive. Since affective symptoms and cognitive functioning are interrelated among older persons, a synergistic effect can be assumed in patients with affective symptoms who also have suffered from child abuse. This study examines the association between a history of child abuse and cognitive performance in such patients.

**Methods:**

Cross-sectional data were collected from the ‘Routine Outcome Monitoring for Geriatric Psychiatry & Science’ project, including 179 older adults (age 60–88 years) with either a unipolar depressive, any anxiety, or somatic symptom disorder referred to specialized geriatric mental health care. A history of physical, sexual, and psychological abuse, and emotional neglect was assessed with a structured interview. Cognitive functioning was measured with three paper and pencils tests (10-words verbal memory test, Stroop Colour-Word test, Digit Span) and four tests from the computerized Cogstate Test Battery (Detection Test, Identification Test, One Card Learning Test, One Back Test). The association between a history of child abuse and cognitive performance was examined by multiple linear regression analyses adjusted for covariates.

**Results:**

Principal component analyses of nine cognitive parameters revealed four cognitive domains, i.e., visual-verbal memory, psychomotor speed, working memory and interference control. A history of child abuse was not associated with any of these cognitive domains. However, when looking at the specific types of child abuse separately, a history of physical abuse and emotional neglect were associated with poorer interference control. A history of physical abuse was additionally associated with better visual-verbal memory.

**Conclusions:**

The association between a history of child abuse and cognitive performance differs between the different types of abuse. A history of physical abuse might particularly be a key determinant of cognitive performance in older adults with a depressive, anxiety, or somatic symptom disorder. Future studies on the impact of these disorders on the onset of dementia should take child abuse into account.

**Trial registration:**

ROM-GPS is registered at the Dutch Trial Register (NL6704 at www.trialregister.nl).

## Background

Child abuse is a major global burden that impairs lifelong mental and physical health with deleterious personal and societal effects. Child abuse can be defined as any type of abuse that is threatening or violent to a child, including abuse or neglect in a physical, emotional, or sexual manner [1]. Worldwide, 25% of adults have been physically abused in their childhood, and 20% of women and 7.7% of men sexually abused, respectively [2]. In the Netherlands it is estimated that 3% of all children are victims of child abuse [3]. Of this reported 3%, the majority experienced emotional neglect (36%), followed by physical neglect (24%). A history of child abuse still has detrimental adverse effects later in life, such as a higher incidence of smoking, obesity, substance abuse and violent behavior or victimization [2, 4]. Moreover, people with a history of child abuse have an increased risk of somatic diseases, especially neurological and musculoskeletal disorders, followed by respiratory, cardiovascular, gastrointestinal, and metabolic disorders [5], as well as mental illnesses [6, 7]. The experience of child abuse remains a risk factor for the (first) onset of depression in later life (> 60 years) [8, 9]. Besides affecting the prevalence, a history of child abuse also plays a role in the clinical characteristics of depressive and anxiety disorders. For instance, adults experiencing depression or anxiety with a history of child abuse have an earlier age of onset, experience more severe symptoms and have a worse treatment response [10]. Whether a history of child abuse is also associated with worse cognitive performance in later life and dementia remains unknown [11].

Among non-psychiatric samples, a meta-analysis has demonstrated that a history of child abuse is associated with worse cognitive performance in adolescents and younger adults [12]. The impact of a history of child abuse on cognitive performance in later life, however, shows a complex picture [13–22]. In the Irish Longitudinal Study on Ageing (*n* = 6912) people who had experienced sexual abuse had a better performance in several cognitive domains despite poorer psychological health compared to people without a history of sexual abuse [13]. In contrast, another study also solely focused on the impact of having experienced sexual abuse found that a history of sexual abuse was associated with worse executive functioning, especially in the presence of an APOE e4 allele [14]. The other studies that examined different types of child abuse identified differential impact of specific types of child abuse or early life adversities on cognitive performance [15–22]. Worse cognitive performance in later life has been shown to be specifically related to the loss of a parent [15–18], physical neglect [19], and having lived in a residential or foster care [20]. A gap in the literature is the lack of studies among older patients with the common mental disorders like depressive, anxiety, and somatic symptom disorders. Since affective symptoms and cognitive functioning are interrelated [21], a synergistic effect can be assumed in these patient groups who also have suffered from childhood abuse. A small study among older patients with mixed depressive and anxiety disorders showed that childhood traumata, particularly physical and sexual events, were associated with worse performance on measures of processing speed, attention, and executive functioning [22]. In a large community-based study, accumulation of adverse childhood events predicted a faster 10-year decline in processing speed, but only among persons with clinically relevant depressive symptoms [23].

One of the underlying mechanisms that causes child abuse to affect the brain and its affective and cognitive functioning, is the neurobiological cascade which entails activation of three stress response systems. The first one is the hippocampus and hypothalamic-pituitary-adrenal (HPA) axis that is involved in glucocorticoid regulation, mainly of cortisol. The second system involved the noradrenergic and adrenaline response to stress. The last and less explored system is the vasopressin-oxytocin stress response. Overactive stress responses caused by the experience of child abuse interfere with neurogenesis and thereby affect structural and functional brain development in a lasting manner [24–26]. The main structural changes are a reduction in the size of the mid-portions of the corpus callosum, impaired development of the left neocortex, hippocampus and amygdala [24, 25]. Functionally, an increased electrical irritability in limbic structures and a reduced functional activity of the cerebellar vermis have been observed [24, 25].

Another underlying mechanism is the impact of experiencing child abuse on the immune system, as low-grade inflammation has been associated with cognitive decline [27]. Two meta-analyses have revealed significant associations between childhood adverse events (such as physical and sexual abuse, parental absence, maternal separation) and the inflammatory markers CRP, IL-6, TNF-a, and fibrinogen [28, 29].

At the molecular level, meta-analysis has shown that a history of child abuse results in epigenetic changes in the fields of HPA-axis functioning and beyond with the strongest impact of total trauma scores, followed by physical and sexual abuse [30].

The objective of the present study is to investigate the association between a history of any type of child abuse and specific types of child abuse on cognitive performance in older patients with either a unipolar depressive, any anxiety, or somatic symptom disorder. Based on the few studies among persons with these disorders or affective symptoms [22, 23], we hypothesize that a history of child abuse is associated with a poorer processing speed, and more executive dysfunction. We secondly hypothesize that, based on the same literature, a history of physical and sexual abuse will most likely yield a negative association with cognitive functioning.

## Methods

### Study design

Baseline data were used from the Routine Outcome Monitoring for Geriatric Psychiatry and Science (ROM-GPS) project [31], an ongoing clinical cohort study in the Northern and central regions of the Netherlands. The ROM-GPS project was set up to examine the effectiveness of the first year of treatment of older patients referred for specialized geriatric mental health care for treatment. Eligible patients were all patients aged 60 years or older who suffered from either a unipolar depressive, any anxiety, or somatic symptom disorder as confirmed by the Mini International Neuropsychiatric Interview Plus (MINI-Plus), a semi-structured psychiatric diagnostic interview [32]. We included patients with a unipolar major depressive disorder (single/recurrent episode), persistent depressive disorder (dysthymia), depressive disorder due to another medical condition, panic disorder, agoraphobia, social anxiety disorder, generalized anxiety disorder, somatic symptom disorder including the specifier with predominant pain, and finally illness anxiety disorder. To meet the current DSM-V criteria, adaptations were made in the MINI-Plus, as this interview was still based on the DSM-IV. The MINI-Plus was administered by well-trained mental health professional independent of the clinical intake team as part of routine clinical care. In addition, these professionals screened global cognitive functioning with the Montreal Cognitive Assessment (MoCA) [33].

Exclusion criteria are 1) an established diagnosis of a neurodegenerative disorder or less than 18 points on the MoCA test, 2) a current or past bipolar or psychotic disorder, 3) a severe substance-use disorder that requires specialized treatment, 4) being physically or mentally too handicapped to administer self-report questionnaires or perform cognitive testing, or 5) insufficient mastery of the Dutch language.

Eligible patients were asked informed consent after oral and written study information, whereafter they received a detailed baseline assessment, which consists of observer- and self-rated questionnaires, a brief physical examination and a cognitive test battery. The baseline assessment took on average three hours and was conducted by well-trained research assistants. This assessment included observer-rated and self-report questionnaires, a brief physical examination, and a cognitive test battery, all in a fixed order starting with the cognitive testing. When patients became too tired, the assessment was spread over two days. At the time of data-extraction, 192 patients were included and had completed the baseline assessment.

The study protocol of ROM-GPS was reviewed by the ethical review board of the University Medical Center Groningen (METc 2014/106), and found to be in line with the Dutch law. ROM-GPS was registered at the Dutch Trial Register (NTR6874 at www.trialregister.nl). The study was conducted in accordance with the ethical principles of the World Medical Association Declaration of Helsinki.

#### Measurements

##### Child abuse

A history of child abuse was assessed with a structured interview, previously used in the Netherlands Mental Health Survey and Incidence Study (NEMESIS) [34] and the Netherlands Study of Depression in Older persons (NESDO) [35]. This interview consists of three sections, i.e., ‘unfavourable social conditions’, such as divorce of parents and foster home placement, ‘negative life events before the age of 16’, which includes four types of child abuse and lastly ‘negative life events after the age of 16’, which represents sexual abuse after the age of 16. For this study, only the second section ‘negative life events before the age of 16’ was evaluated as the other two sections do not cover child abuse. In this second section, participants were asked if, and if yes how frequent ranging from 1 (once) to 5 (very frequently), and by whom they have been abused in their childhood. Four types of abuse were inquired about: emotional neglect, psychological abuse, physical abuse, and sexual abuse. Inquiry about each type, started with a brief clarification to the subject of the kind of experiences meant by that term. Emotional neglect was described as not being listened to at home, your experiences or problems being ignored or not feeling like you can count on your parents for attention and support. Psychological abuse includes being cursed at, unjustified punishments, being disadvantaged with brothers and sisters and being blackmailed. Physical abuse includes being kicked or beaten, but also other types of physical abuse. Sexual abuse includes being sexually touched or being forced to sexually touch somebody against your will. In this study, participants were considered to have experienced child abuse if they had a frequency score of 1 or more on any of the types of abuse. We also considered the different types of abuse, physical, sexual, and psychological abuse and emotional neglect, as separate determinants. In this case, participants were considered to have experienced the type of abuse in question if they scored a frequency of 1 or more for that specific subtype of trauma.

##### Cognitive functioning

Cognitive functioning was assessed with traditional paper and pencil tests as well as computerized tests. We administered three paper and pencil tests, i.e., the 10-words test (a modified version of the Auditory Verbal Learning Test), a shortened version of the Stroop Colour-Word test and Digit Span test from the Wechsler Adult Intelligence Scale (WAIS III). These tests have sufficient to good test-retest and inter-rater reliability and the specific versions have been described extensively [36]. In addition, the Detection Test, Identification Test, One Card Learning test, and One Back Test from the computerized Cogstate Test Battery were administered (see http://www.cogstate.com) [31].

Nine cognitive measures were obtained; five based on the paper and pencil tasks, i.e., processing speed (number of seconds for Stroop tasks I & II together), working memory (number of correct answers on Digit Span forward & backward together), immediate recall (total number of correct recalls on five presentations of 10-words test), delayed recall (total number of correct recalls from single delayed recall of 10-words test), and interference control (proportion of extra time for Stroop tasks III compared to mean time on tasks I and II)), and four on the Cogstate battery, i.e., psychomotor functioning (Detection Test), attention (Identification Test), working memory (One Back Test) and visual learning (One Card Learning Test). Two of these measures (i.e., processing speed and interference control) had to be transformed into their inverse (1/x), to make their distributions more normal. Finally, all measures were standardized into Z-scores and recoded so that higher scores indicate better cognitive functioning. To examine if the cognitive measures assessed separate or related cognitive domains, a principal component factor analysis (PCA) was performed, with oblique rotation (oblimin) because the final factors were expected to be intercorrelated. Factors were extracted based on eigenvalues > 0.7 [37], observation of the scree plot (a significant drop in the plot marks a significant reduction in percentage of variance explained by the next factor compared to the already included factors), interpretability of the factors, and coverage of all cognitive measures by the factors. Factor scores were calculated by multiplying the Z-scores of the measures by their factor score coefficient in the final rotated factor solution.

A total of 140/192 (72.9%) participants had complete data on all nine cognitive measures. The PCA on these 140 participants with complete data (KMO measure of sampling adequacy: *p* = .669) showed that three factors had an eigenvalue > 1, while the scree-plot suggested a four-factor solution. This four-factor solution was also clinically meaningful as it included a separate factor representing interference control (an aspect of executive functioning), which was not covered by solutions with less factors. Factor 1 was labelled as ‘visual-verbal memory’, factor 2 as ‘psychomotor speed’, factor 3 as ‘working memory’ and factor 4 as ‘interference control’. The four factors together explained 73.6% of the variance of the original nine cognitive tasks. Table 1 presents the rotated factor solution. Pearson’s correlation coefficient of factor 1 with 3 was .33 and .15 with factor 2. The correlations between the other factors were less than .10.

**Table 1:** Factor scores and solution derived by PCA with direct oblimin rotation on 140 participants

	Factor 1	Factor 2	Factor 3	Factor 4
*Paper and pencil measures:*				
- Processing speed	.361	**.547**	**.544**	−.108
- Working memory	.149	.015	**.781**	.387
- Verbal memory- Immediate	**.887**	.257	.346	.085
- Verbal memory- Delayed	**.898**	.191	.144	.108
- Interference control	.182	−.015	.160	**.940**
*Cogstate Battery:*				
- Psychomotor functioning	−.145	**.879**	−.136	.097
- Attention	−.159	**.896**	.012	−.052
- Working memory-simple	.360	.113	**.802**	−.100
- Visual Learning	**.546**	−.164	**.456**	−.025
	Visual-verbal memory	Psychomotor speed	Working memory	Interference control

Of the 52 participants with missing data on one or more of the cognitive measures, we were able to estimate factor scores 40 persons, by estimating their missing measure(s) from the available ones. Of these, 10 persons had missing data for one or two of the nine cognitive tests, and 30 persons had multiple missing scores on the Cogstate battery but none on the paper and pencil measures. This resulted in a final dataset of 180 participants with data on the four cognitive factors (of which for one of these participants the data on the experience of childhood abuse was missing).

##### Covariates

As potential confounders on the association between a history of child abuse and cognitive functioning, we assessed demographic characteristics (age, sex, level of education), lifestyle characteristics, symptom severity of the affective disorder, somatic comorbidities, and prescribed drug use.

The level of education was categorized as low (no education, only elementary school, or a 4-year secondary school), medium (completed 5 or 6-year secondary education, or a vocational degree), or high (completed a 4-year college, or university degree).

Lifestyle characteristics included smoking (classified as currently, ever, or never), alcohol use (based on the Alcohol Use Disorders Identification Test (AUDIT) as abstinent (score of 0), recreational (score 1–6), or harmful (score 7–40) [38], and physical activity as the total MET-minutes a week based on the International Physical Activities Questionnaire (IPAQ) short form [39]. A MET-minute represents the amount of energy expended carrying out physical activity relative to that of resting.

The severity of the psychiatric disorders was assessed with three self-report questionnaires well-validated for use in older people, i.e., depressive symptoms with the 30-item Inventory of Depressive Symptoms (IDS-SR), anxiety with the Geriatric Anxiety Inventory (GAI), and severity of hypochondria with the Whitely Index (WI) [40–42]. In our sample, the Cronbach’s alpha for the IDS, GAI, and WI were 0.88 (good), 0.91 (excellent), and 0.76 (acceptable), respectively.

Somatic comorbidity was assessed with a well-validated self-report questionnaire that covered the most prevalent chronic diseases in the Netherlands, including 1) obstructive lung disease such as asthma and COPD, 2) cardiac disease, 3) peripheral arterial disease, 4) hypertension, 5) diabetes mellitus, 6) cerebrovascular accident, 7) osteoarthritis and/or rheumatoid arthritis and 8) cancer [43]. The number of chronic diseases was assessed, with a range of 0–8.

For each type of medication used by the participant, the name, dosage and frequency of intake was asked. To correct for antidepressants and benzodiazepines as confounders, two dichotomous variables were created that indicated whether or not the participant currently uses an antidepressant or took benzodiazepines at least 4 days a week.

##### Statistical analyses

To assess differences in baseline characteristics between abused and non-abused participants, chi-squared tests were applied for categorical variables and independent t-tests for continuous variables (as all were normally distributed).

Linear regression analyses were performed to examine the association between a history of any child abuse, as well as the different types of child abuse, and cognitive functioning as the dependent variable. For each cognitive factor separately, two models were tested: model 1 with any type of child abuse (yes/no) as independent variable, and model 2 with each specific type of child abuse entered simultaneously as independent variables. All regression models were performed with and without covariates. For each model, we present the unstandardized coefficient (B, including its standard error), the standardized coefficient (β), and the effect size for child abuse (f^2^). An f^2^ of 0.02 is generally considered a small effect, whereas an f^2^ of either 0.15 or 0.30 are considered a medium or large effect.

Since the associations with specific types of child abuse in history might be biased due to comparison with participants with both no childhood abuse as well as participants with a history of another type of child abuse, sensitivity analyses were conducted excluding patients with a history of other types of abuse from the reference group.

Significance was set at *p* < 0.05, with the statistical analyses being carried out with IBM SPSS version 26.

## Results

### Study sample

Of the 192 participants included in the second stage of the ROM-GPS study, 179 (93.2%) participants had valid data on both a history of child abuse and cognitive functioning. Participants with missing data did not significantly differ from participants with complete data on any of the descriptive variables (lowest *p*-value was .163 for sufficient physical activity).

Of the 179 participants, 103 (57.5%) experienced child abuse before the age of 16 years. Of these abused participants, 45 (43.7%) were physically abused, 43 (41.7%) were sexually abused, 61 (59.2%) were psychologically abused, and finally 81 (78.6%) were emotionally neglected in their youth. These numbers do not add up to 100% as 71/103 (68.9%) participants with a history of child abuse report more than one type of abuse.

As shown in Table 2, patients with a history of any type of child abuse were significantly older (*p* = .038), as well as more severely depressed and anxious (both *p* < .001).

**Table 2:** Baseline characteristics of the study sample (*n* = 179)

		History of child abuse		
Characteristics:		None (*n* = 76)	Present (*n* = 103)	Statistics^**a**^
*Socio-demographics:*				
• Age (years)	mean (SD)	70.3 (6.2)	68.4 (5.6)	t = 2.1, df = 177, *p* = .038
• Female sex	n (%)	35 (46.1)	59 (57.3)	Chi^2^ = 2.2, df = 1, *p* = .137
• Level of education:				
- Lower	n (%)	27 (35.5)	38 (36.9)	
- Medium	n (%)	29 (38.2)	32 (31.1)	Chi^2^ = 1.2, df = 2, *p* = .562
- Higher	n (%)	20 (26.3)	33 (32.0)	
*Physical & cognitive functioning:*				
• Number of chronic diseases	mean (SD)	2.0 (1.5)	2.0 (1.4)	t = 0.2, df = 177, *p* = .854
• Cognitive performance (MoCA)	mean (SD)	28.5 (1.2)	28.7 (1.4)	t = −1.2, d = 174, *p* = .220
*Psychopathology:*				
• Depressive symptom severity (IDS)	mean (SD)	30.6 (11.9)	37.1 (10.9)	t = −3.8, df = 177, *p* < .001
• Anxiety symptoms (GAI)	mean (SD)	10.3 (4.8)	13.0 (4.8)	t = −3.6, df = 175, *p* < .001
• Hypochondriasis (WI)	mean (SD)	4.7 (3.0)	5.3 (2.9)	t = −1.4, df = 176, *p* = .171
• Any depressive disorder^b^	n (%)	58 (76.3)	80 (77.7)	Chi^2^ < 0.1, df = 1, *p* = .831
• Any anxiety disorder^b^	n (%)	38 (50.0)	52 (50.5)	Chi^2^ < 0.1, df = 1, *p* = .949
• Any somatic symptom disorder^b^	n (%)	25 (32.9)	32 (31.1)	Chi^2^ = 0.1, df = 1, *p* = .795
• Antidepressant drug use	n (%)	48 (67.6)	58 (60.4)	Chi^2^ = 0.9, df = 1, *p* = .340
• Benzodiazepine use	n (%)	23 (32.4)	30 (31.3)	Chi^2^ = 0.0, df = 1, *p* = .875
*Lifestyle characteristics:*				
Smoking:				
° Never	n (%)	20 (26.3)	26 (25.2)	
° Formerly	n (%)	37 (48.7)	53 (51.5)	Chi^2^ = 0.1, df = 2, *p* = .933
° Currently	n (%)	19 (25.0)	24 (23.3)	
Alcohol use:				
° Abstinent	n (%)	29 (28.2)	30 (29.1)	
° Recreational use	n (%)	35 (46.1)	56 (54.4)	Chi^2^ = 1.7, df = 2, *p* = .429
° Problematic use	n (%)	12 (15.8)	17 (16.5)	
• Sufficient physical activity	n (%)	56 (75.7)	72 (70.6)	Chi^2^ = 0.6, df = 1, *p* = .454

^a^ Student’s T-test or Chi^2^-test

^b^ Numbers do not add to 100% as 85/179 (47.5%) patients had comorbid psychiatric disorders between the three main diagnostic groups

### Association between a history of child abuse and cognitive functioning

Table 3 presents the associations between a history of child abuse overall as well as the specific types of abuse and the four cognitive factors. We did not find a significant association between a history of child abuse and any of the four cognitive factors. Regarding the specific types of child abuse, a history of physical abuse was associated with better visual-verbal memory and, on the other hand, worse interference control (B(SE) = 0.31 (0.15), ß = 0.13, *p* = .048, and B(SE) = − 0.55 (0.18), ß = -0.23, *p* = .002, respectively). A history of emotional neglect was also associated with worse interference control (B(SE) = − 0.35 (0.16), ß = -0.17, *p* = .032). These associations remained statistically significant when adjusted for covariates.

**Table 3:** A history of child abuse^a^ as a determinant of cognitive functioning by multivariate linear regression

	Unadjusted				Adjusted^**b**^			
	B (SE)	β	***p***-value	f^**2**^	B (SE)	β	***p***-value	f^**2**^
**Visual-verbal memory (factor 1)**								
*Model 1:*								
• Any child abuse (yes/no)	0.37 (0.15)	0.18	.016	0.03	0.21 (0.14)	0.10	.153	0.01
*Model 2:*								
• Physical abuse (yes/no)	0.40 (0.17)	0.17	.020	0.08	0.31 (0.15)	0.13	.048	0.04
• Sexual abuse (yes/no)	0.53 (0.17)	0.23	.002		0.31 (0.16)	0.13	.058	
• Psychological abuse (yes/no)	0.29 (0.16)	0.14	.068		0.25 (0.15)	0.12	.089	
• Emotional neglect (yes/no)	0.22 (0.15)	0.11	.149		0.09 (0.14)	0.05	.517	
**Psychomotor speed (factor 2)**								
*Model 1:*								
• Any child abuse (yes/no)	0.24 (0.14)	0.13	.089	0.02	0.24 (0.15)	0.13	.111	0.02
*Model 2:*								
• Physical abuse (yes/no)	0.22 (0.16)	0.10	.175	0.02	0.18 (0.17)	0.08	.288	0.01
• Sexual abuse (yes/no)	−0.05 (0.16)	−0.02	.780		−0.05 (0.17)	−0.03	.753	
• Psychological abuse (yes/no)	0.20 (0.15)	0.10	.174		0.19 (0.16)	0.10	.220	
• Emotional neglect (yes/no)	0.15 (0.14)	0.08	.276		0.15 (0.15)	0.08	.320	
**Working memory (factor 3)**								
*Model 1:*								
• Any child abuse (yes/no)	0.10 (0.15)	0.05	.508	< 0.01	0.11 (0.15)	0.06	.447	< 0.01
*Model 2:*								
• Physical abuse (yes/no)	0.15 (0.17)	0.07	.362	0.06	0.10 (0.16)	0.04	.546	0.04
• Sexual abuse (yes/no)	0.15 (0.17)	0.07	.366		0.11 (0.16)	0.05	.503	
• Psychological abuse (yes/no)	−0.18 (0.15)	−0.09	.240		−0.17 (0.15)	−0.08	.256	
• Emotional neglect (yes/no)	0.20 (0.15)	0.10	.173		0.22 (0.14)	0.11	.130	
**Interference control (factor 4)**								
*Model 1:*								
• Any child abuse (yes/no)	−0.08 (0.16)	−0.04	.607	< 0.01	−0.23 (0.17)	−0.11	.172	0.01
*Model 2:*								
• Physical abuse (yes/no)	−0.45 (0.18)	−0.19	.010	0.06	−0.55 (0.18)	−0.23	.002	0.07
• Sexual abuse (yes/no)	0.08 (0.18)	0.03	.658		0.05 (0.19)	0.02	.773	
• Psychological abuse (yes/no)	−0.08 (0.16)	− 0.04	.621		−0.19 (0.17)	− 0.09	.271	
• Emotional neglect (yes/no)	−0.19 (0.16)	−0.09	.222		−0.35 (0.16)	−0.17	.032	

^a^ Separate analyses for the determinants in model 1 regarding the history of any type of abuse (yes/no), and model 2 regarding the four types of child abuse (yes/no) entered simultaneously

^b^ Adjusted for age, sex, level of education, number of somatic diseases, antidepressant drug use, benzodiazepine use, depressive symptom severity, anxiety level, hypochondriacal beliefs, alcohol use, smoking and physical activity

### Sensitivity analyses of the significant associations

As in the original analyses, significant associations with worse interference control were found when participants with a history of physical abuse were compared to those with no abuse (ß = -.28, *p* = .006), and when participants with a history of emotional neglect were compared to those with no abuse (ß = -.18, *p* = .049) for interference control. However, the association with visual-verbal memory was no longer found (ß = .18, *p* = .059) for participants with a history of physical abuse compared to those with no abuse. All these associations were adjusted for the covariates.

## Discussion

In this study, we showed that in older adults with a depressive, anxiety, or somatic symptom disorder, only a history of specific types of child abuse before the age of 16 years (i.e., physical abuse and emotional neglect) were associated with poorer cognitive functioning (i.e., interference control). Effect-sizes, however, were small and the different types of abuse had different effects on the cognitive domains, varying from negative to positive. This could be the reason why no significant association between a history of any child abuse and the cognitive domains could be found. Chances are that the different types of abuse cancel each other’s effects out when added together. This explanation is in line with previous studies that found no association between cumulative measures of child abuse and cognitive performance, while associations were found with specific types of child abuse (e.g., [17, 19, 20]).

### Impact of types of child abuse

Experience of physical abuse and emotional neglect in childhood were associated with decreased interference control, a type of executive functioning, which is in line with our hypothesis. This negative association between a history of child abuse and cognitive functioning in older adults is consistent with multiple studies [14, 19, 22, 23] and can be explained by the theories on the stress response and inflammatory response that follow the experience of child abuse, as previously discussed [24–30]. A systematic review showed that neuroimaging findings in depression and anxiety like reduced hippocampal volume and amygdala hyperreactivity were more pronounced among adult patients with a history of child abuse compared to depressed or anxious patients without a history of child abuse, as well as were epigenetic modifications and genetic polymorphisms [44]. Since executive functioning represents higher-order cognitive abilities relevant for many lower-order cognitive domains, even small effects may have clinically relevant impact when facing cognitive decline in later life.

We found no negative association between a history of child abuse in general and processing speed, in contrast to our hypothesis and several previous studies in younger as well as in older people with depressive symptoms [12, 23].

Also in contrast with our hypothesis was the positive association between a history of physical abuse and visual-verbal memory, but this might be a chance finding as it did not pass the sensitivity analysis. Nonetheless, there are a few studies that also showed a positive association between a history of child abuse and cognitive functioning in later life [13, 16]. In The Irish Longitudinal Study on Ageing on almost 7000 persons aged 50 years or older, childhood sexual abuse was associated with better global cognition, memory, executive functioning and processing speed, despite poorer psychological health in this group compared to those without a history of sexual abuse [13]. In another study of 1282 community-dwelling older adults aged 65 years or older, childhood adversities, such as sharing of parental problems and loss of a parent, were negatively associated with verbal fluency and memory, while a history of physical and sexual abuse were positively associated with higher performance on verbal fluency and memory [16]. These positive associations might be explained by between person differences in the impact of child abuse on either mental health and cognitive functioning. Adequate coping strategies and a healthy lifestyle, for example, may increase cognitive reserve [45]. Following this reasoning, our selection of patients with affective disorders may have resulted in an overrepresentation of patients resilient for negative cognitive effects of child abuse in later life. Another explanation might be related to the fact that hypercortisolism after early life trauma often results in hypocortisolism at middle and older age [46]. As neurotoxic effects of high cortisol levels are shown to be persistent in middle and old age in the form of cognitive decline [47], it is possible that trauma-related hypocortisolism is neuroprotective. A final explanation could be that mild level of noradrenergic mediated hyperarousal, due to child abuse (as classically observed in PTSD patients) [48] may improve cognitive performance. This latter explanation, however, could not be explored as we did not assess PTSD symptomatology in our study.

### Methodological considerations

To our knowledge, this is the first study on the association between a history of child abuse and cognitive functioning in older adults with a mixed group of internalizing psychiatric disorders (depressive, anxiety, or somatic symptom disorders), while controlling for a large variety of confounders. Most previous studies investigated anxiety and depression separately, while this study looks at them together. This is clinically relevant as these groups of mental disorders have the highest prevalence rates in later life and high mutual comorbidity rates, and longitudinal studies have shown diagnostic instability over time between these groups of disorders [49, 50]. For proper interpretation, however, some limitations should be addressed.

First, selection bias may have diluted the possible overall negative association between a history of child abuse and cognitive functioning since A) patients with severe cognitive impairment (MoCA < 18 points) were excluded, B) patients with a history of child abuse may be underrepresented because they may be less likely to engage in research due to emotion-regulation problems, and C) survival bias due to excess mortality rates at younger ages of people with a history of child abuse. Secondly, only the negative association between a history of physical abuse and interference control would remain significant when a Bonferroni correction for multiple testing is applied (.05/16 = .003). Thirdly, our study is limited by its cross-sectional design. Although child abuse occurs by definition before cognitive decline in old age, the retrospective assessment of child abuse may result in underreporting due to memory impairment or overreporting due to a depressed mood. On the other hand, empirical data suggest that retrospective assessment of child abuse is unlikely to be affected by psychopathology, [51, 52] and the test-retest reliability of retrospective self-reports on child abuse in older adults is moderate to good and neither influenced by age nor cognitive functioning [53].

## Conclusions

The negative association between a history of physical abuse and interference control, a type of executive functioning, in older adults with an affective disorder contributes to a growing body of evidence that a history of child abuse has even an impact on cognitive performance in old age. Such knowledge is important as child abuse has been hypothesized as a risk factor for dementia as currently known risk factors cannot explain the full extent of the onset of dementias [11]. Moreover, the experience of child abuse should be considered as an overall underlying mechanism in studies examining the impact of either anxiety disorders and/or depressive disorders on the onset of dementia [54, 55]. Future studies on potentially mediating mechanisms underlying this association should also take type and severity of abuse into account.

## Data Availability

Researchers interested in checking or using the data of the ROM-GPS project, can contact the principal investigator prof. R.C. Oude Voshaar (r.c.oude.voshaar@umcg.nl). Data for future studies will only be made available in collaboration with the principal investigator and only in case a good research question is formulated, including a hypothesis and an elaborated statistical plan.

## Abbreviations

ROM-GPS: Routine Outcome Monitoring for Geriatric Psychiatry and Science
CRP: C-reactive protein
IL-6: Interleukin 6
TNF-a: Tumor Necrosis Factor alfa
HPA-axis: Hypothalamic Pituitary Adrenal axis
MINI-plus: Mini International Neuropsychiatric Interview Plus
MoCA: Montreal Cognitive Assessment
METc: Medisch-Ethische Toetsingscommissie (‘Ethics Committee)
NTR: Netherlands Trial Register
PCA-: Principal Component Analysis
AUDIT: Alcohol Use Disorder Identification Test
IPAQ: International Phyiscal Activity Questionnaire
IDS-SR: Inventory of Depressive Symptoms – Self-Report
GAI: Geriatric Anxiety Inventory
WI: Whitley Index
